# Ultra-High-Frequency ECG in Cardiac Pacing and Cardiac Resynchronization Therapy: From Technical Concept to Clinical Application

**DOI:** 10.3390/jcdd11030076

**Published:** 2024-02-23

**Authors:** Uyên Châu Nguyên, Jesse H. J. Rijks, Filip Plesinger, Leonard M. Rademakers, Justin Luermans, Karin C. Smits, Antonius M. W. van Stipdonk, Frits W. Prinzen, Kevin Vernooy, Josef Halamek, Karol Curila, Pavel Jurak

**Affiliations:** 1 Department of Cardiology, Maastricht University Medical Center+, Cardiovascular Research Institute Maastricht (CARIM), 6229 HX Maastricht, The Netherlands; jesse.rijks@mumc.nl (J.H.J.R.); justin.luermans@mumc.nl (J.L.); twan.van.stipdonk@mumc.nl (A.M.W.v.S.); kevin.vernooy@mumc.nl (K.V.); 2 Department of Physiology, Maastricht University Medical Center+, Cardiovascular Research Institute Maastricht (CARIM), 6229 HX Maastricht, The Netherlands; karin.smits@maastrichtuniversity.nl (K.C.S.); frits.prinzen@maastrichtuniversity.nl (F.W.P.); 3 Institute of Scientific Instruments of the Czech Academy of Sciences, 61200 Brno, Czech Republic; fplesinger@isibrno.cz (F.P.); josef@isibrno.cz (J.H.); jurak@isibrno.cz (P.J.); 4 Department of Cardiology, Catharina Ziekenhuis, 5623 EJ Eindhoven, The Netherlands; nard.rademakers@catharinaziekenhuis.nl; 5 Cardiocenter, Third Faculty of Medicine, Charles University and University Hospital Kralovske Vinohrady, Srobarova, 1150/50, 10034 Prague, Czech Republic; curilakarol@seznam.cz

**Keywords:** cardiac resynchronization therapy, conduction system pacing, ultra-high frequency, electrocardiography, electrical dyssynchrony

## Abstract

Identifying electrical dyssynchrony is crucial for cardiac pacing and cardiac resynchronization therapy (CRT). The ultra-high-frequency electrocardiography (UHF-ECG) technique allows instantaneous dyssynchrony analyses with real-time visualization. This review explores the physiological background of higher frequencies in ventricular conduction and the translational evolution of UHF-ECG in cardiac pacing and CRT. Although high-frequency components were studied half a century ago, their exploration in the dyssynchrony context is rare. UHF-ECG records ECG signals from eight precordial leads over multiple beats in time. After initial conceptual studies, the implementation of an instant visualization of ventricular activation led to clinical implementation with minimal patient burden. UHF-ECG aids patient selection in biventricular CRT and evaluates ventricular activation during various forms of conduction system pacing (CSP). UHF-ECG ventricular electrical dyssynchrony has been associated with clinical outcomes in a large retrospective CRT cohort and has been used to study the electrophysiological differences between CSP methods, including His bundle pacing, left bundle branch (area) pacing, left ventricular septal pacing and conventional biventricular pacing. UHF-ECG can potentially be used to determine a tailored resynchronization approach (CRT through biventricular pacing or CSP) based on the electrical substrate (true LBBB vs. non-specified intraventricular conduction delay with more distal left ventricular conduction disease), for the optimization of CRT and holds promise beyond CRT for the risk stratification of ventricular arrhythmias.

## 1. Introduction

Cardiac resynchronization therapy (CRT) is a guideline-recommended therapy for patients with dyssynchronous heart failure (HF). CRT significantly impacts patients’ trajectories and is associated with left ventricular (LV) reverse remodeling and improved clinical outcomes [1,2,3,4].

Identifying electrical dyssynchrony is crucial for patient selection and optimization in CRT. To define electrical dyssynchrony, it is essential to understand its meaning in the context of ventricular conduction disturbances. Invasive electrophysiological evaluations have revealed that a typical left bundle branch block (LBBB), which represents the ideal substrate for CRT, is characterized by a uniform right-to-left activation with delayed ventricular conduction due to an increased duration of transseptal activation and slow LV myocardial activation [5,6].

In current clinical practice, an assessment of electrical dyssynchrony is primarily conducted using 12-lead electrocardiogram (ECG)-derived QRS duration and morphology. QRS duration, however, remains a crude measure of electrical dyssynchrony and does not provide information about the underlying electrical conduction disturbance. QRS morphology provides more detailed information about the underlying electrical conduction; however, the commonly used LBBB definitions can be prone to subjectivity and may be influenced by the chosen definition and heart–torso geometry [7,8].

**Figure 1:**
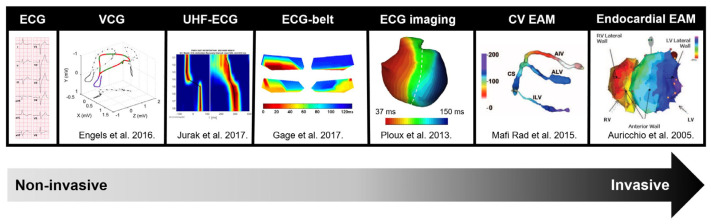
Overview of measurement methods used for electrical activation modified with permission from Nguyen et al. [28], along with the frequently used parameters for detecting electrical dyssynchrony. Abbreviations: CV = coronary venous, EAM = electro-anatomic mapping, ECG = electrocardiogram, UHF-ECG = ultra-high-frequency ECG, VCG = vectorcardiography.

Over the past decade, various alternative methods of ventricular dyssynchrony assessment have been proposed. These range from simple options such as vectorcardiography [9,10,11] and ultra-high-frequency ECG (UHF-ECG) to more advanced techniques like ECG-belt [12,13,14,15], ECG-imaging [16,17,18,19,20,21] and electro-anatomic mapping [6,22,23,24,25,26] (Figure 1).

The UHF-ECG technique is unique due to its ability to perform dyssynchrony analyses instantaneously with real-time visualization, making it particularly attractive for implementation in standard clinical practice [27]. This paper will comprehensively discuss the physiological background of using higher frequencies in ventricular conduction and explore the translational evolution of UHF-ECG in cardiac pacing and CRT, from technical concept development to its clinical application in various forms of cardiac pacing and CRT.

## 2. High Frequencies and Ventricular Activation

Variations in wavefront propagation speed throughout the cardiac cycle are manifested through differences in the frequency content of activation and repolarization waves on the ECG. Under physiological conditions, the P wave typically falls within the range of 5–30 Hz (reflecting an atrial propagation velocity of ~1 m/s), the QRS complex spans 5–50 Hz (corresponding to a His–Purkinje system propagation velocity of 1–4 m/s), and the T wave is predominantly found within the 0–10 Hz range, indicating a relatively slow repolarization spread [29,30].

Einthoven noted that “an EKG recorded with a galvanometer, with a deflection time of about 0.01 s, does not practically differ from the same EKG recorded with a galvanometer with an infinitely small deflection time”. This suggests that frequencies greater than 100 Hz have negligible contributions to the ECG signal [31] Scher and Young conducted frequency analyses of the ECG in seventeen healthy individuals and eight not-further-specified patients [32]. They observed that frequencies above 90 Hz contributed to less than two percent of the QRS amplitude, leading to the similar conclusion that frequencies exceeding 100 Hz do not significantly contribute to the ECG. While it therefore may be justifiable to use the commercial frequency range of <150 Hz in normal individuals, the presence of higher frequencies has been linked to ventricular pathologies.

High-frequency notching has been associated with the presence of patchy fibrosis in the necropsies of patients after myocardial infarction [33]. Remarkably, these high-frequency components may already manifest during ischemia [34,35] The presence of high-frequency components (in the range of 150–250 Hz) was observed in canine studies after inducing ischemia through the occlusion of the left anterior descending coronary artery, and they have been proposed as a more sensitive marker than traditional ST-segment elevation [36,37]. The existence of high-frequency components up to 700 Hz in the QRS complex has been concordantly associated with ventricular enlargement, hypertrophy, and myocardial scar [38,39,40]. High-frequency components (particularly above 70 Hz) were also observed by the group of Josephson in endocardial electrograms and associated with reentrant ventricular tachycardia [41,42,43]. As a result, the signal-averaged ECG method was introduced, employing a bandpass of 40–250 Hz to detect late ventricular potentials [44].

Before the introduction of UHF-ECG, only limited research had been conducted in the field of dyssynchrony and frequency content. The influence of LBBB on the signal-averaged ECG was investigated by the group of El-Sherif in 48 patients with intrinsic LBBB on the surface ECG and in 39 patients with a normal surface QRS duration who underwent right ventricular (RV) pacing-induced LBBB. Using a filter setting of 25–250 Hz, LBBB was characterized by a prolongation of the duration of signals <40 microvolts and fragmented signals in the terminal portion of the filtered QRS [45].

## 3. Rationale and Conceptual Studies on UHF-ECG

**Figure 2:**
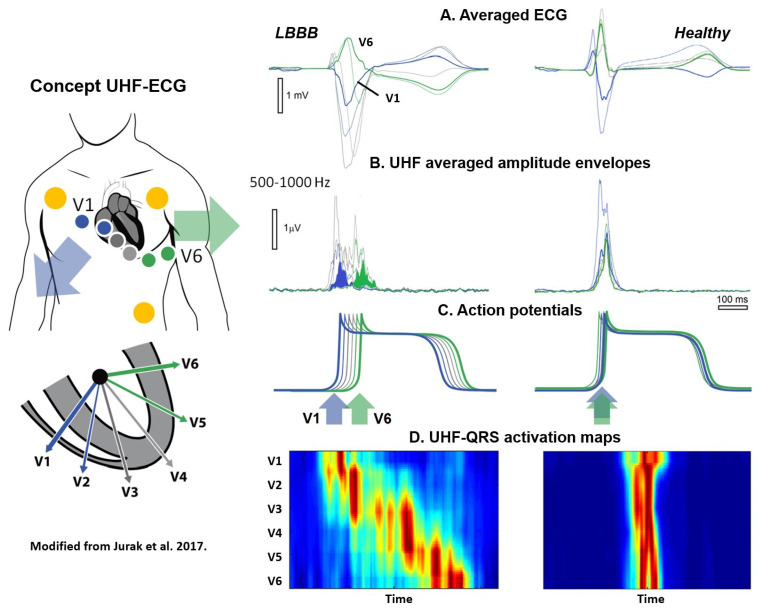
Original concept of UHF-ECG modified with permission from Jurak et al. [46]. Representative UHF-ECG during a LBBB (**left**) and normal (**right**) ventricular activation. A. Averaged UHF-ECG computed from multiple heart beats. B. Averaged UHF amplitude envelopes for 500–1000 Hz. C. Schematic interpretation of myocardial cell action potentials. D. Corresponding UHF-ECG visualization of activation per precordial lead. Red indicates the time (horizontal axis) and location (vertical axis) when most myocardial cells are activated simultaneously. In the initial studies, data from six precordial leads were utilized. In the current application, up to eight precordial leads can now be employed to assess the inferolateral wall.

Past high-frequency ECG studies predominantly concentrated on ischemia, neglected the time domain, employed single leads, and utilized a restricted frequency range of up to 250 Hz. The UHF-ECG technique was first introduced by Jurak et al. in a proof-of-concept study on seventeen patients prior and post CRT implantation as a more refined method for assessing electrical dyssynchrony (Figure 2) [46,47]. Standard ECG systems traditionally record within the bandpass ranges of 0.05–100 Hz or 0.50–150 Hz with six precordial leads and three limb leads. The current UHF-ECG technique records ECG signals with a high sampling rate and a band width of up to 1500 Hz. The ECG is analyzed in 16 frequency bands (width 100 Hz, step 50 Hz, range 150–1050 Hz) from eight precordial leads (V1–V8) [27].

To create a broad-band QRS complex (UHF-QRS), the average of sixteen normalized median (over multiple beats) amplitude envelopes is computed and visualized as a color map for each lead. A UHF-QRS duration at 50% of its amplitude is used to compute the local depolarization time for each precordial lead. Ventricular electrical delay is calculated as the maximum difference between the center of mass of UHF-QRS (local depolarization times) between leads V1–V8 [46]. The Brno laboratory is widely recognized in the field of signal analysis for their development of the free signal analysis software SignalPlant. UHF Solver and VDI Vision software were specifically developed for UHF-ECG data processing [48,49].

The rationale behind the UHF-ECG approach is that the steep gradients in cell membrane potential, resulting from the change in the sodium ion current (phase 0 of the action potential), represent a unique source of UHF oscillations. The UHF-QRS can be interpreted as a histogram of the distribution of UHF oscillations in both time (horizontal time axis) and location (precordial leads). While UHF transmitters operate synchronously in a healthy heart, it is evident that this is not the case in a dyssynchronous heart, leading to a broader UHF oscillation histogram and subsequently more delay between ventricular segments in the precordial leads [46]. In the initial UHF-ECG paper by Jurak et al., UHF-ECG ventricular electrical delay with a cut-off value exceeding 50 ms identified a reduction of 10% or more in LVESV for all CRT recipients [46].

Besides detecting local activation times, the UHF technique also holds the potential to evaluate intramural activation of the ventricles. Jurak et al. demonstrated that high-frequency ECG imaging accurately reflects intramural activation using an ex vivo model. In this experimental setup, two Langendorff-perfused pig hearts were suspended in a human torso-shaped tank. UHF-ECG imaging was performed with surface tank electrodes and validated with epicardial sock and plunge electrodes in the heart [50].

After the initial conceptual studies, Plesinger et al. conducted a retrospective validation of the UHF-ECG technique in a large patient cohort from the Multicenter Automatic Defibrillator Implantation Cardiac Resynchronization Therapy (MADID-CRT) trial [2]. Fully automated ventricular electrical delay was computed using the UHF-ECG approach from 10 min recordings of digital 12-lead ECGs obtained at 1 kHz. A total of 676 patients with LBBB, 113 with right bundle branch block (RBBB), and 160 with intraventricular conduction delay (IVCD) were included, as the computational approach requires a sufficient number of digital sinus beats for accuracy. A ventricular electrical delay of ≥31 ms was significantly associated with a higher risk of HF or death in the LBBB patients, whereas these associations were only borderline significant for IVCD patients and not significant for RBBB patients [51].

**Figure 3:**
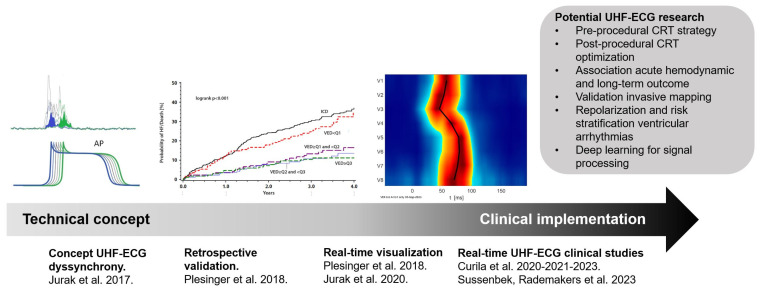
The translational journey of UHF-ECG, from technical concept [46] retrospective validation [2] real-time visualization [27] and implementation in clinical studies. Abbreviations: CRT = cardiac resynchronization therapy, UHF-ECG = ultra-high-frequency ECG.

While these early studies explored the UHF-ECG concept using retrospective data, the implementation of the technique in clinical practice accelerated after the development of a real-time application. This application facilitated the processing of UHF-ECG recordings, enabling instant analysis and providing real-time output of ventricular depolarization maps. This advancement allowed for the practical use of UHF-ECG during implantation and optimization procedures in clinical practice (Figure 3) [27].

## 4. Real-Time UHF-ECG in Clinical Studies

Since its validation in the MADIT-CRT population, UHF-ECG-derived ventricular electrical delay has been utilized as a measurement of dyssynchrony in multiple clinical studies [52,53,54,55,56,57,58], especially in exploring the ventricular activation patterns in different approaches of conduction system pacing (CSP) [53,54,55,56,58]. CSP consists of different novel pacing techniques, more specifically, His bundle pacing (HBP) and left bundle branch area pacing (LBBAP) [59]. Both techniques have been introduced as more physiologic alternatives to anti-bradycardia pacing and biventricular CRT (BiV-CRT) [60,61,62,63,64].

The most physiological form of pacing is theoretically provided by HBP, as there is complete recruitment of the conduction system to both ventricles [65,66,67,68]. UHF-ECG-derived ventricular electrical dyssynchrony and the mean depolarization time of all precordial leads were analyzed to study activation patterns during HBP and (para-Hisian) myocardial pacing by Curila et al. [56]. Both were compared to normal atrioventricular conduction in 46 patients with a pacing indication due to bradycardia and no baseline bundle branch block. Ventricular electrical delay and the mean activation time of all precordial leads were comparable between HBP and normal atrioventricular conduction, whereas (para-Hisian) myocardial pacing showed a significant increase in both parameters when compared to normal atrioventricular conduction and HBP [56]. UHF-ECG thus confirmed that HBP induces a similar electrophysiological activation as during normal intrinsic activation.

LBBAP is a different CSP approach, where the pacing lead is placed transeptally at the left side of the interventricular septum. It comprises LV septal myocardial pacing (LV septal pacing; LVSP) and additional conduction system capture (left bundle branch pacing; LBBP) [59,69,70]. UHF-ECG was used to study the differences between both types of LBBAP. Sixty-eight patients with a pacing indication due to bradycardia were treated with LBBAP. It was shown that LBBP preserves left ventricular synchrony best, but at the cost of increase interventricular dyssynchrony. LVSP produces less interventricular dyssynchrony than LBBP, but at the cost of slightly longer LV lateral wall activations.

Ventricular dyssynchrony and its reduction are of great interest in patients with heart failure, and is an aim of the research by using UHF-ECG in the last few years. Ventricular depolarization patterns were compared between CSP-CRT and conventional BiV-CRT in 80 patients [58]. Both CSP-CRT and BiV-CRT significantly reduced ventricular dyssynchrony in these patients with LBBB. Moreover, it was shown that ventricular electrical delay and mean precordial depolarization times were significantly shorter during CSP-CRT compared to BiV-CRT, suggesting that CSP-CRT reduces ventricular dyssynchrony to a greater extent than BiV-CRT [58].

Also, clinical data showed that HBP and LBBAP show promising results as an alternative way of delivering CRT (CSP-CRT) [64,68]. In a recent observational multicenter study comparing clinical outcomes between CSP-CRT and BiV-CRT involving 1778 patients, CSP-CRT demonstrated a greater reduction in paced QRS duration, an improved LV ejection fraction, and lower mortality or HF-related hospitalization rates compared to BiV-CRT (20.8% vs. 28%; *p* < 0.001) [71].

An overview of the key studies on different pacing strategies using UHF-ECG is provided in Table 1.

## 5. UHF-ECG in Relation to Other Methods for Dyssynchrony Assessment

**Figure 4:**
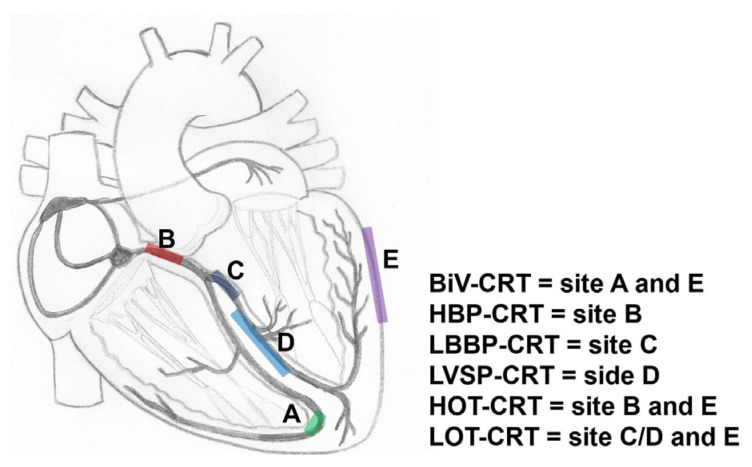
Schematic overview of various approaches of CRT. BiV = biventricular pacing; HOT-CRT = His-optimized CRT; LBBP = left bundle branch pacing; LOT-CRT = left bundle branch area-optimized CRT.

In the current clinical practice, electrical dyssynchrony is mainly assessed using the QRS duration and morphology from the standard 12-lead ECG. In the last decade, various alternative measures of ventricular dyssynchrony have been suggested (Figure 4), spanning from straightforward options near the ECGs that summarize electrical activation to more high-resolution techniques, such as electro-anatomic mapping [8]. Within the spectrum of electrical dyssynchrony methods, the data acquisition of the UHF-ECG is very closely associated with the standard ECG. The practical difference is that up to eight precordial leads are preferentially recorded, and the UHF-ECG parameters are computed from multiple heartbeats with a higher sampling rate and bandwidth compared to the standard ECG.

Ventricular electrical delay, determined from UHF-ECG in 676 LBBB patients from the MADID-CRT trial, predicted the primary endpoint of HF or death more effectively than QRS duration from a standard ECG (*p* < 0.001 vs. *p* < 0.007). Interestingly, there was a significant but only moderate correlation between ventricular electrical delay and QRS duration (R = 0.50). Dichotomized ventricular electrical delay did not demonstrate predictive value in the non-LBBB population [51].

The vectorcardiographic (VCG) QRS area, defined as the time integral of the QRS complex in three orthogonal leads from either the true Frank vectorcardiogram or the reconstructed vectorcardiogram, has shown significant promise as a measure for predicting outcomes in CRT. The relationship between the UHF-ECG ventricular electrical delay and QRS area has been investigated by Halamek et al. in a subset of the MADID-CRT population. Interestingly, UHF-ECG demonstrated a stronger correlation with the QRS area than QRS duration (R = 0.473 and R = 0.154, respectively) [72].

The viability of incorporating visualization analysis software into speckle tracking echocardiography and its comparison with UHF-ECG was demonstrated in a study involving 17 patients. The comparison between speckle tracking imaging and UHF-ECG revealed a novel parameter: the time delay between electrical and mechanical onset. However, the application of speckle tracking echocardiography is not feasible in all patients due to the necessity for good image quality, a limitation not encountered by UHF-ECG [46].

Since UHF-ECG, in principle, measures the timing of the spatial activation of the ventricles, studies that relate ventricular hemodynamics and electrical dyssynchrony using echocardiography or arterial blood pressure measurements will be of great interest.

## 6. Future Prospects

### UHF-ECG for Determining Resynchronization Approaches and Optimization

**Figure 5:**
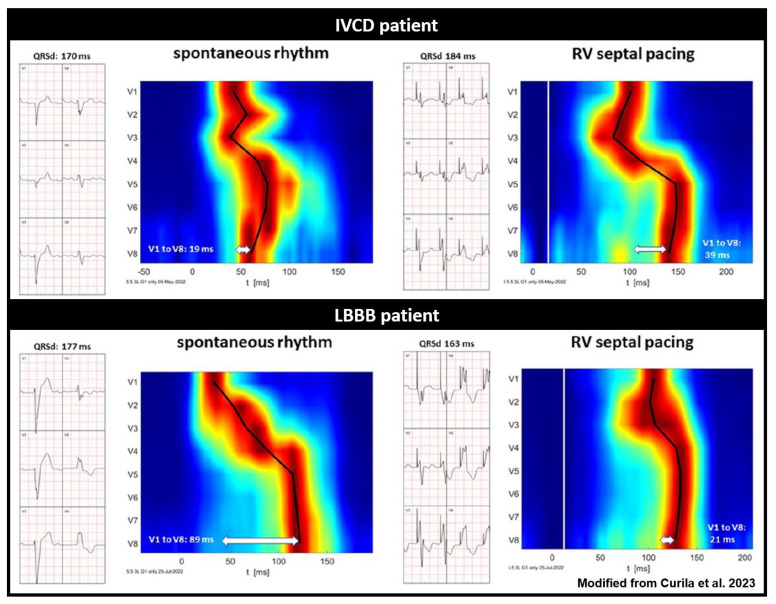
Standard ECG and UHF-ECG activation maps of two patients with wide QRS complexes, illustrating the potential of UHF-ECG in distinguishing between true LBBB and IVCD (modified with permission from Curila et al.) [78]. Red indicates the time (horizontal axis) and location (vertical axis) when most myocardial cells are activated simultaneously. The upper panel reflects a patient with IVCD, and the lower panel depicts a patient with true LBBB activation. It is noteworthy that RV septal pacing prolongs QRS duration and ventricular electrical delay in the IVCD patient, while it reduces QRS duration and ventricular electrical delay in the true LBBB patient.

HBP can properly resynchronize only in very proximal intra-Hisian blocks, where LBBAP can correct the block when the block is located intra- or infra-Hisian, but still within the proximal parts of the left bundle branch [65,73]. With a more distal myocardial conduction delay due to underlying substrate [74] a combination of both BiV-CRT and HBP or LBBAP might be necessary. These therapies are referred to as HOT-CRT (His bundle-optimized CRT) or LOT-CRT (left bundle branch-optimized CRT) [73,75,76]. Where the HBP or LBBAP pacing leads will be in lieu of the RV lead; an additional LV lead will be placed via the coronary sinus. A pre-implantation assessment of the site of the block (proximal or distal) can be useful for patient selection and planning. UHF-ECG can potentially show the differences in ventricular activation patterns caused by a proximal and more distal conduction system disease by studying the activation patterns during intrinsic rhythm and RV septal pacing (Figure 5) [77,78]. Validation studies on the ability of UHF-ECG with invasive mapping for the detection of different types of conduction systems are therefore highly relevant.

**Figure 6:**
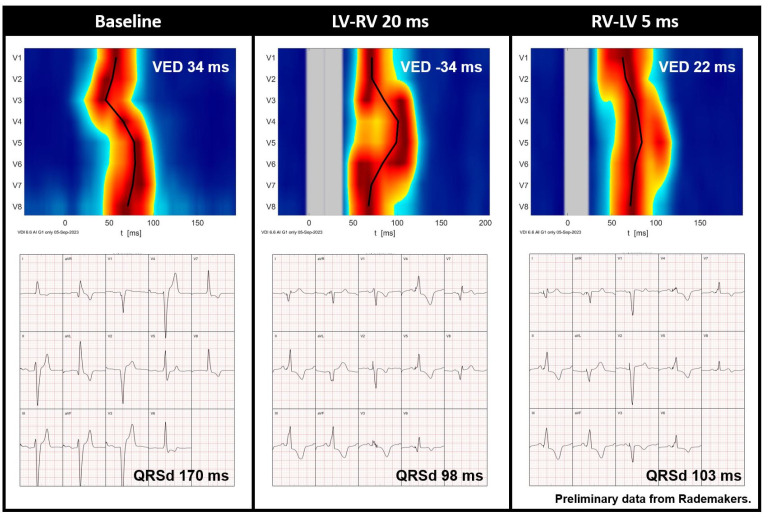
Preliminary data from Rademakers et al. demonstrating the UHF-ECG activation map and averaged ECG of a CRT recipient before implantation and during various VV interval settings. Red indicates the time (horizontal axis) and location (vertical axis) when most myocardial cells are activated simultaneously. It is noteworthy that QRS duration is relatively short during an LV-RV of 20 ms, whereas the UHF-ECG reveals dyssynchronous activation. In contrast, an RV-LV of 5 ms results in more synchronous activation (ventricular electrical delay, 22 ms). Abbreviations: QRSd = QRS duration, VED = ventricular electrical delay.

In addition to selecting the right type of resynchronization, UHF-ECG, similar to other dyssynchrony approaches, may play a role in the optimization of resynchronization (Figure 6) [47]. Although optimization may not be necessary in all CRT recipients [4,12], it may aid symptomatic patients with moderate or poor disease modification after CRT. Additional studies have shown that UHF-ECG can display instant electrical activation during various types of pacing, including RV pacing [52] HBP [56] LBBAP [53,54,55], and BiV-CRT [58].

The future of UHF-ECG can also be seen in the simplification of implantation procedures and the availability of CSP techniques. One may assume that it is not so important to precisely define the pacing type, but rather to monitor ventricular dyssynchrony and try to minimize it. The extents to which optimization with UHF-ECG is related to acute hemodynamic response and improves patient outcome on long term still need to be investigated.

## 7. Technical and Clinical Research beyond CRT

The (re)introduction and enhancement of high-frequency ECG measurements can stimulate additional exploration into the frequency and time domain characteristics of electrical activation at both thoracic and intracardiac levels under various physiological conditions and specific pathologies. UHF-ECG captures a broad spectrum of frequencies. For instance, it would be intriguing to examine the impact of these higher frequencies on the computation of ventricular dyssynchrony, especially in the case of LBBB.

In its current form, UHF-ECG can generate real-time depolarization maps within 1–3 min during implantation [27,57]. Future improvements may involve updating the QRS detection method [79] and pacemaker stimulus elimination technique using deep learning models, along with a more specified frequency range tailored to each clinical application. These enhancements to the instant visualization approach could potentially result in a shorter analysis time and more precise depolarization localization.

Examining high-frequency components could have implications for the study of ventricular arrhythmia. In a recent proof-of-concept study with 60 participants, UHF-ECG was used to evaluate the degree of fragmentation. The severity of fragmentation, quantified by the number of peaks, showed a correlation with arrhythmia risk status across all participants. This correlation persisted when comparing patients at high risk to those at low risk for inherited diseases, highlighting the potential of UHF-ECG in stratifying sudden death risk in individuals with inherited cardiac conditions [80].

While higher frequencies offer insights into pathophysiological characteristics during depolarization, lower frequencies may unveil repolarization traits, given the prevalence of low frequencies in the T wave. With the widespread adoption of CSP, studying repolarization in relation to ventricular arrhythmias becomes particularly intriguing. The occurrence of sustained ventricular tachycardia (VT) or ventricular fibrillation (VF) in patients undergoing either CSP-CRT or BiV-CRT was recently investigated in a multicenter observational study involving 1778 patients [81]. CSP-CRT was associated with a lower incidence of VT/VF, (4.2% vs. 9.3%, *p* < 0.001) and lower occurrence of VT storm (0.8% vs. 2.5%, *p* = 0.013) compared to conventional BiV-CRT. This observation was also present in a subanalysis involving patients who received CRT for primary prevention (CSP-CRT vs. BiV-CRT: 3.2% vs. 7.3%, *p* = 0.007) [81]. One might hypothesize that more physiological activation (rather than the non-physiological epicardial to endocardial activation during BiV-CRT) could induce a more physiological repolarization, subsequently inducing more antiarrhythmic effects.

## 8. UHF-ECG Limitations

Compared to other commercial methods for dyssynchrony assessment, the UHF-ECG technique is accessible, but it does require either a dedicated UHF-ECG device or integration as a software module into other commercial devices. The amplitude of the signals recorded is in the range of micro Volts, and for these reasons, care must be taken to ensure that the recording location is free of interfering background noise signals.

Currently, the VDI UHF-ECG device (VDI Technologies) is fully available as a research experimental device. At the same time, the certification process is underway, which should enable full commercial clinical use. The expense of recording a 12- or 14-lead ECG is very low and comparable to a regular ECG. The demands on the operator are minimal, and UHF-ECG maps and numerical parameters (activation times, dyssynchrony, local activation duration) are provided automatically without human intervention.

## 9. Conclusions

This review delves into the physiological foundation of employing higher frequencies in ventricular conduction and traces the translational evolution of UHF-ECG in cardiac pacing and CRT. Since the advent of standard ECG systems, research on the frequency spectrum of ventricular activation has been somewhat overlooked. Although high-frequency components during activation were studied half a century ago, they have rarely been explored in the context of dyssynchrony. The UHF-ECG technique records ECG signals from eight precordial leads, incorporating the time domain as well. Following the initial conceptual studies, the UHF-ECG technique was further optimized to enable the instant visualization of ventricular activation. UHF-ECG has been utilized for patient selection in CRT and the evaluation of ventricular activation during CSP. It has the potential to play a role in determining a tailored resynchronization approach by assessing the underlying ventricular conduction and in the further optimization of CRT settings. Furthermore, UHF-ECG shows promise beyond CRT, such as in risk stratifying for ventricular arrhythmias.

## Figures and Tables

**Table 1:** Key studies on different pacing strategies using UHF-ECG.

Study	Year	Patients No.	Pacing Indication	Pacing Strategy Studied	Main Finding
Jurak et al. [46]	2017	17	CRT	BiV-CRT	Introducing UHF-ECG technique for identification differences in electrical activation.
Plesinger et al. [51]	2018	949	CRT	BiV-CRT	LBBB patients with a high baseline UHF-ECG ventricular electrical delay benefited most from CRT.
Curila et al. [56]	2020	46	Bradycardia	HBP	Both selective and non-selective HBP, but not myocardial pacing, preserve ventricular electrical synchrony as measured using UHF-ECG.
Curila et al. [52]	2021	51	Bradycardia	RVP/non-selective HBP/RBBP	RV inflow tract pacing produces better ventricular synchrony than other RV pacing locations. Concomitant capture of His bundle or proximal RBB along with adjacent myocardium results in the most synchronous ventricular activation.
Curila et al. [53]	2021	68	Bradycardia	LBBP/LVSP	LBBP preserves physiological LV depolarization but increases interventricular dyssynchrony. LVSP prolongs LV lateral wall depolarization but preserves interventricular dyssynchrony to the same level as HBP.
Curila et al. [55]	2021	57	Bradycardia	LBBP/LVSP	In patients with bradycardia, LVSP in close proximity to LBB resulted in better interventricular synchrony than non-selective LBBP and selective LBBP, and did not significantly prolong the depolarization of the left ventricular lateral wall.
Curila et al. [54]	2023	75	Bradycardia	HBP/LBBP/RVSP	Although anodal LBBP improved ventricular synchrony and the depolarization duration of the septum and RV compared to unipolar non-selective LBBP, the resultant ventricular depolarization was still less physiological than during HBP.
Sussenbek et al. [58]	2021	80	CRT	LBBAP-CRT/BiV-CRT	Both BiV-CRT and LBBAP significantly reduce ventricular dyssynchrony in CRT patients with LBBB. The left bundle branch area pacing is associated with more physiological ventricular activation.

Abbreviations: HBP = His bundle pacing; RVP = right ventricular pacing; RBBP = right bundle branch pacing; LBBP = left bundle branch pacing; LVSP = left ventricular septal pacing; RVSP = right ventricular septal pacing; LBBAP = left bundle branch area pacing; BiV-CRT = biventricular cardiac resynchronization therapy; RV = right ventricle; RBB = right bundle branch; LV = left ventricle; LBB = left bundle branch; CRT = cardiac resynchronization therapy.
